# Dual-Phase Severity Grading of Strawberry Angular Leaf Spot Based on Improved YOLOv11 and OpenCV

**DOI:** 10.3390/plants14111656

**Published:** 2025-05-29

**Authors:** Yi-Xiao Xu, Xin-Hao Yu, Qing Yi, Qi-Yuan Zhang, Wen-Hao Su

**Affiliations:** College of Engineering, China Agricultural University, 17 Qinghua East Road, Haidian District, Beijing 100083, China; 2022307140224@cau.edu.cn (Y.-X.X.); 2022307140212@cau.edu.cn (X.-H.Y.); 19970780888@163.com (Q.Y.); qiyuanzhang@cau.edu.cn (Q.-Y.Z.)

**Keywords:** deep learning, strawberry angular leafspot disease, computer vision, severity classification, smart agriculture

## Abstract

*Phyllosticta fragaricola*-induced angular leaf spot causes substantial economic losses in global strawberry production, necessitating advanced severity assessment methods. This study proposed a dual-phase grading framework integrating deep learning and computer vision. The enhanced You Only Look Once version 11 (YOLOv11) architecture incorporated a Content-Aware ReAssembly of FEatures (CARAFE) module for improved feature upsampling and a squeeze-and-excitation (SE) attention mechanism for channel-wise feature recalibration, resulting in the YOLOv11-CARAFE-SE for the severity assessment of strawberry angular leaf spot. Furthermore, an OpenCV-based threshold segmentation algorithm based on H-channel thresholds in the HSV color space achieved accurate lesion segmentation. A disease severity grading standard for strawberry angular leaf spot was established based on the ratio of lesion area to leaf area. In addition, specialized software for the assessment of disease severity was developed based on the improved YOLOv11-CARAFE-SE model and OpenCV-based algorithms. Experimental results show that compared with the baseline YOLOv11, the performance is significantly improved: the box mAP@0.5 is increased by 1.4% to 93.2%, the mask mAP@0.5 is increased by 0.9% to 93.0%, the inference time is shortened by 0.4 ms to 0.9 ms, and the computational load is reduced by 1.94% to 10.1 GFLOPS. In addition, this two-stage grading framework achieves an average accuracy of 94.2% in detecting selected strawberry horn leaf spot disease samples, providing real-time field diagnostics and a high-throughput phenotypic analysis for resistance breeding programs. This work demonstrates the feasibility of rapidly estimating the severity of strawberry horn leaf spot, which will establish a robust technical framework for strawberry disease management under field conditions.

## 1. Introduction

As a globally cultivated fruit crop valued for its rich content of sugars, vitamins, and minerals, strawberry (*Fragaria × ananassa*) has garnered significant research attention in agronomy, genomics, and nutrition sciences due to its susceptibility to pathogens that severely impact its yield and nutritional quality [1,2]. In strawberry production, disease is a major threat to quality and yield. Common strawberry diseases include powdery mildew, gray mold, anthracnose, and root rot [3]. Among these, angular leaf spot is one of strawberries’ most common and significant diseases. Its symptoms are manifested in the early stage of the disease; there are many black spots on the edges of the leaves, followed by water-soaked, red-brown irregular lesions on the lower surface. In the later stage, the lesions gradually expand and leave combined lesions on the leaf surface [4]. If the prevention and control of strawberry angular leaf spot disease is not timely, it will cause growth point necrosis and plant death, resulting in a decline in quality, reduction in yield, and economic losses. The timely detection of diseases and real-time precision spraying are effective means to control diseases. The method of assessing disease severity traditionally relies on the trained naked eye. However, this method is laborious, costly, time-consuming, and prone to human error. Therefore, it is urgently required to develop a more effective and high-throughput method for field assessment of the disease [5,6].

Computer vision has been widely implemented in agricultural research, notably in crop phenotyping acquisition and evaluation of disease [7,8,9]. R. Meena Prakash et al. [10] proposed a crop disease detection and classification method based on transfer learning and an optimized convolutional neural network (CNN). Similarly, R. Thyagaraj et al. [11] proposed a classification method of plant leaf diseases based on an improved support vector machine (SVM). Through image preprocessing, segmentation, and feature extraction, combined with an SVM classifier, the high-precision classification of plant leaf diseases was realized, and the test accuracy rate reached 95.0%. Additionally, traditional computer vision techniques remain relevant; for instance, S. Arivazhagan et al. [12] utilized K-means clustering to segment diseased leaf regions, achieving a 94.0% accuracy in classifying plant leaf diseases using texture features. More recently, J. G. A. Barbedo [13] combined K-means clustering with SVM to identify plant diseases from lesion spots in field conditions, reporting accuracies up to 95.0%, highlighting the enduring utility of classical methods in practical agricultural settings

In recent years, with the rapid development of deep learning, innovative technologies have been continuously integrated into the field of image recognition. Deep learning has the advantages of a fast recognition speed and high accuracy [14,15]. The target detection algorithm for plant diseases based on deep learning can be divided into a two-stage model represented by the region-based convolutional network (R-CNN) series and a one-stage detection model represented by the YOLO series [16]. These techniques have been widely used in the identification of pests and diseases. Shafik et al. [17] proposed a new hybrid convolutional neural network (Inception–Xception CNN) for the identification of plant diseases. The network combines the advantages of Inception and Xception architecture and achieves the high-precision recognition of plant leaf diseases through multi-scale feature extraction and deep supervised learning. The experimental results show that the model has achieved excellent performance on multiple plant disease datasets. Khan et al. [18] proposed a real-time apple leaf disease detection system based on deep learning. By improving the Faster-RCNN model, combining the convolutional block attention module (CBAM) and the ultra-lightweight dynamic upsampling operator (DySample), the detection accuracy and real-time performance of the model are significantly improved. The system has been successfully applied to the real-time detection of apple leaf diseases. Roy et al. [19] proposed an enhanced YOLOv4 model based on DenseNet. This model achieves the efficient detection of mango growth stages in complex environments by optimizing feature propagation and reuse mechanisms and combining an improved PANet structure to retain fine-grained information. Cardellicchio et al. [20] designed a single-stage detector based on improved YOLOv5 to identify the phenotypic traits of tomato plants (such as nodes, fruits, and flowers) and showed a high detection accuracy in complex datasets characterized by small targets, a high similarity, and tight color matching. Olisah et al. [21] proposed a multi-input convolutional neural network ensemble classifier (MCE), which is optimized by a pre-trained VGG16 model and can effectively identify the subtle features of blackberry maturity. Chen et al. [22] proposed an improved strawberry maturity detection algorithm based on the CES-YOLOv8 network structure. By replacing part of the C2f module in the YOLOv8 model trunk with the ConvNeXt V2 module and introducing an ECA attention mechanism, its feature representation ability is further improved. The experimental results show that the accuracy of the improved model in complex environments is improved by 4.8%.

This study innovatively proposed an improved dual-phase strawberry angular leaf spot disease classification method based on YOLOv11 for segmenting strawberry leaves and OpenCV threshold segmentation for segmenting the lesion part. The specific objectives of this study are as follows: (1) a proposal for a dual-phase diagnostic framework is presented, integrating YOLOv11-based leaf segmentation with threshold segmentation-based disease spot segmentation, which achieves a significant reduction in background interference while maintaining computational efficiency; (2) an optimized YOLOv11 model achieves the pixel-level segmentation of strawberry leaves and accurately classifies healthy versus diseased ones; (3) an OpenCV threshold segmentation algorithm efficiently detects lesions; (4) a quantitative grading system is established based on the ratio of diseased to total leaf area, validated with established datasets; (5) a PyQt5 (V5.15.11) software application incorporates the enhanced YOLOv11-CARAFE-SE model for practical disease diagnosis and phenotypic analysis in precision breeding.

As far as we know, this study represents the first application of a cascaded segmentation framework—integrating YOLOv11 with threshold segmentation—to the severity grading of strawberry angular leaf spot, enabling automated disease severity assessments in complex field scenarios.

## 2. Results

### 2.1. Model Training

**Figure 1:**
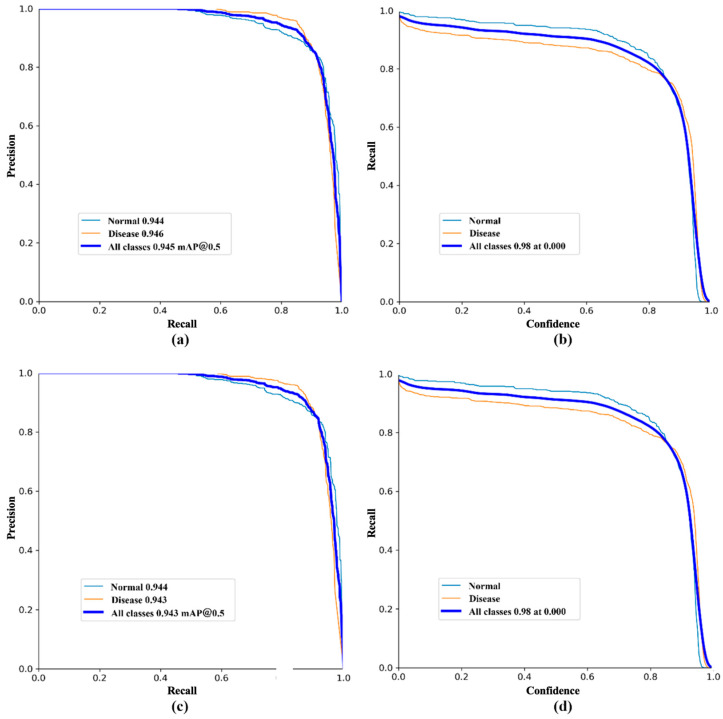
Performance evaluation curves of the YOLOv11-CARAFE-SE model, demonstrating a high accuracy and reliability. (**a**) Box precision–recall curve; (**b**) box recall–confidence curve; (**c**) mask precision–recall curve; (**d**) mask recall–confidence curve.

**Figure 2:**
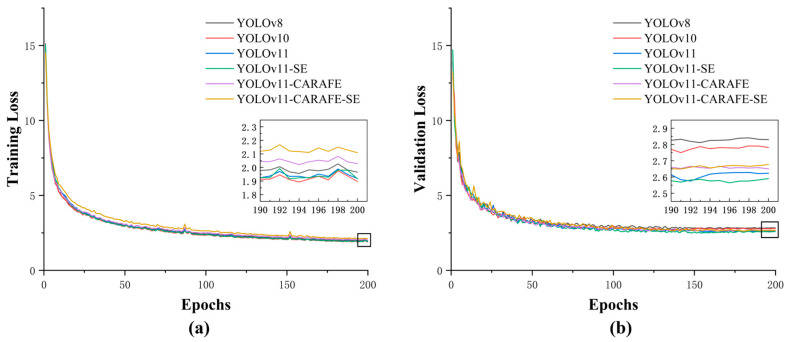
Recognizing training loss curves (**a**) and validation loss curves (**b**) for strawberry leaf segmentation using the six proposed YOLOv11-based models. The loss for the improved YOLOv11-CARAFE-SE model demonstrates a clear tendency towards stabilization in the later training epochs. A magnified view of epochs 190–200 is included to allow for closer inspection of this stabilization.

To compare the performance of various models, this study developed six primary segmentation models based on annotated strawberry disease images: YOLOv8, YOLOv10, YOLOv11, YOLOv11-SE, YOLOv11-CARAFE, and YOLOv11-CARAFE-SE, with YOLOv11-CARAFE-SE serving as the enhanced model. Figure 1 illustrates the performance evaluation curves of the YOLOv11-CARAFE-SE model. Specifically, the figure includes the precision–recall curve and recall–confidence curve for box segmentation, as well as the precision–recall curve and recall–confidence curve for mask segmentation. The recall–confidence relationship demonstrates robust performance, maintaining a recall of 98% for all classes even at the highest confidence thresholds, thereby indicating reliable predictions with a high certainty. Specifically, YOLOv8 and YOLOv10 were utilized to evaluate performance improvements within YOLOv11, while YOLOv11-SE and YOLOv11-CARAFE were employed to assess the contributions of the SE and CARAFE modules to the YOLOv11 model. Figure 2a presents the training loss values of each model on the strawberry diseased leaf segmentation task as the number of epochs increases, and Figure 2b depicts the corresponding validation loss values as a function of epochs on the validation set. To facilitate a more granular analysis of the learning dynamics during the terminal phase of training, a magnified view of both training and validation loss curves, specifically from epoch 190 to 200, is presented. Observation of this magnified segment reveals that while the training loss of the proposed YOLOv11-CARAFE-SE model is marginally elevated compared to some baseline models, this discrepancy is minimal. Critically, this is accompanied by a competitive validation loss and, most importantly, a demonstrably superior mAP. This confluence of metrics suggests that the enhanced model has likely acquired more generalizable feature representations and exhibits a greater robustness against overfitting to the training data. Overall, the loss functions of all the models gradually decrease with increasing epochs, ultimately stabilizing; however, after stabilization, the differences in loss values among the models become relatively minor.

### 2.2. Ablation Experiments

To further assess the effectiveness of the individual modules within the YOLOv11-CARAFE-SE model, this study selected the YOLOv11n model as the baseline for comparison experiments. Figure 2 illustrates the loss curves of various models, demonstrating that all have converged after 200 epochs. An ablation study was conducted on the test set by sequentially integrating each module into the baseline network, in conjunction with a detailed analysis of the dataset. The corresponding results are summarized in Table 1. The comparative data indicate that the YOLOv11 model yields significant improvements relative to the YOLOv8 model across multiple performance metrics. In comparison with the original YOLOv11n model, the inclusion of the SE module notably enhances recall, albeit with a potential reduction in precision; importantly, it results in a significant improvement in mAP@0.5. Conversely, the CARAFE module exerts a minimal influence on recall, while enhancing precision and markedly boosting mAP@0.5. Ultimately, because of integrating the SE and CARAFE modules, the enhanced YOLOv11-CARAFE-SE model preserves a stable precision, with box precision increasing slightly from 88.1% to 88.3%, while mask precision exhibits a negligible decrease from 88.4% to 88.2%. In contrast, recall is significantly improved, with box recall rising from 86.0% to 87.2% and mask recall from 86.3% to 87.3%. Moreover, mAP@0.5 demonstrates a marked enhancement, with box mAP@0.5 increasing from 91.8% to 93.2% and mask mAP@0.5 from 92.1% to 93.0%. Additionally, the inference time is reduced from 1.3 ms to 0.9 ms, and the computational load decreased from 10.3 GFLOPS to 10.1 GFLOPS.

### 2.3. Impact of Different Attention Mechanisms on the Model

This study incorporated three attention mechanisms—SE, CBAM, and Context Aggregation—into the original YOLOv11 model and conducted comparative experiments, as presented in Table 2. All the models were evaluated using identical parameter configurations and under consistent conditions. According to the experimental results, when all the models were constrained to a computational load of 10.4 GFLOPS, each attention mechanism enhanced the box mAP@0.5. Notably, the improvement was most pronounced with the SE mechanism, whereas both CBAM and Context Aggregation resulted in a decrease in mask mAP@0.5. Overall, the experimental findings indicate that the SE attention mechanism demonstrates superior performance in this scenario.

### 2.4. Effects of Different Upsampling Methods on the Model

The original YOLOv11 model employs nearest neighbor interpolation for the upsampling. The introduced CARAFE and DySample modules are enhancements built upon nearest neighbor upsampling and bilinear interpolation, respectively. To enable a more rigorous comparison, the upsampling method in the original YOLOv11 model was altered from nearest neighbor interpolation to bilinear interpolation. According to the experimental results presented in Table 3, the enhanced model incorporating the CARAFE module demonstrated the most significant performance improvements across all the evaluated metrics—achieving an mAP of 93.1%—while also reducing GFLOPS to a minimum of 10.1.

### 2.5. Performance of the Improved Model in Strawberry Angular Leaf Spot Leaf Segmentation

**Figure 3:**
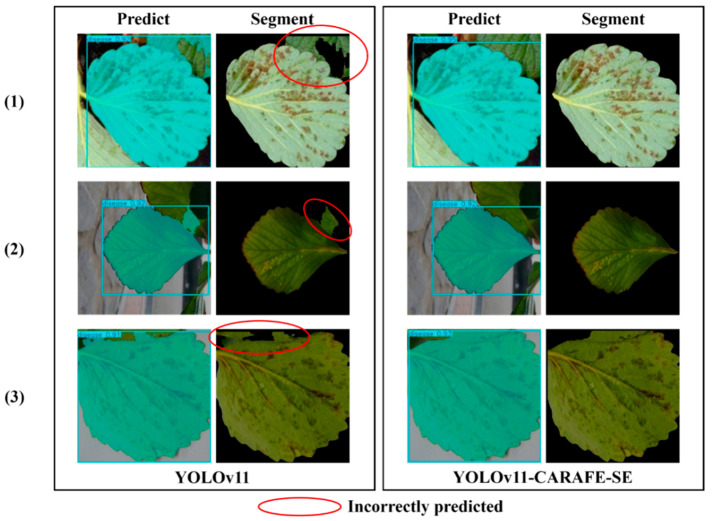
The three segmentation examples comparing the performance of YOLOv11 and YOLOv11-CARAFE-SE in strawberry angular leaf spot leaf segmentation, demonstrating how YOLOv11-CARAFE-SE correctly segments areas where the baseline YOLOv11 model errs. The disease area with the red circle is a false positive (FP), since it was predicted but has no corresponding ground truth.

To validate the effectiveness of the improved model, this study compared the detection outcomes of the original YOLOv11 model and the optimized YOLOv11-CARAFE-SE model under real-world conditions. Figure 3 presents three representative detection outcomes as examples. Although strawberry leaves were mis-segmented by the original YOLOv11 model, they were correctly segmented by the improved YOLOv11-CARAFE-SE model. The experimental results indicated that in complex natural backgrounds, the YOLOv11-CARAFE-SE model more accurately extracts the features of various diseases, achieving a higher segmentation accuracy and prediction confidence compared to the original YOLOv11 model. By incorporating the CARAFE and SE modules into YOLOv11, their synergistic effect enhanced the model’s feature extraction capabilities, thereby significantly improving its detection performance.

### 2.6. Disease Spot Segmentation Based on OpenCV and Disease Severity Classification

**Figure 4:**
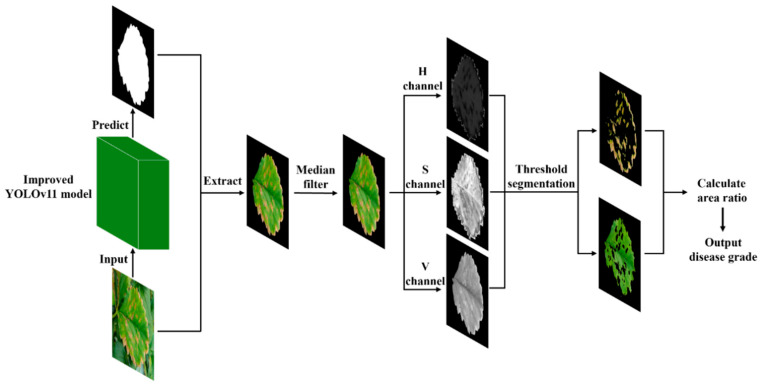
The complete process for calculating strawberry disease severity based on YOLOv11 and OpenCV.

Figure 4 illustrates the flowchart of the disease spot segmentation process implemented using OpenCV. After validating the method’s effectiveness, this study performed disease spot segmentation on a training set comprising 2225 leaf images, resulting in images that exclusively display the diseased portions, along with their corresponding area ratio data. Given that data augmentation was applied to simulate real-world variations in shooting angles, lighting intensity, and other factors, and considering that segmentation was performed only on the leaf with the highest prediction confidence for each captured image, it is possible that the leaf identified as having the highest prediction confidence may differ between the original image and its four augmented versions. To ensure the reliability of the disease severity classification thresholds, inconsistent experimental data were excluded from the analysis. This procedure yielded 485 high-consistency datasets that were used to establish the severity classification thresholds.

**Figure 5:**
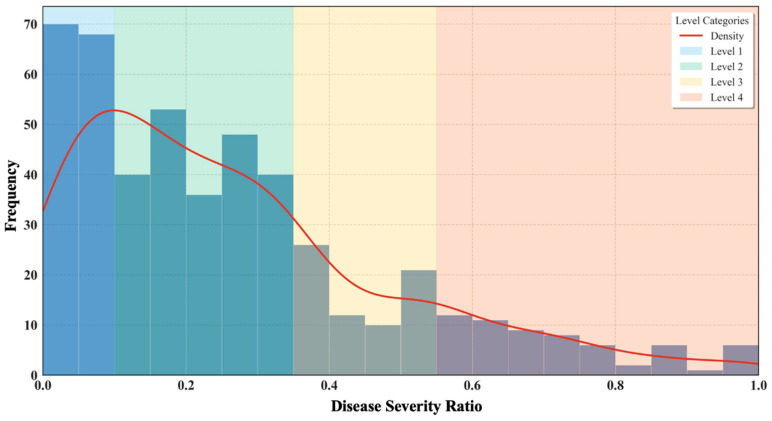
The frequency distribution chart of the disease area ratio and the classification of severity levels: the disease area ratio has a range of [0, 1] with an interval of 0.05, and thresholds of 0.1, 0.35, and 0.55 are used to divide the data into four levels.

A histogram, illustrated as Figure 5, was constructed in this study to illustrate the frequency distribution of the proportion of lesion area to total leaf area (disease severity ratio). The disease severity ratio was defined within the range of [0, 1], with an interval of 0.05. Additionally, to provide a clearer visualization of the data distribution trend, a kernel density estimation (KDE) curve was overlaid, with the bandwidth set to 0.8. Analysis of the KDE curve indicated a decreasing trend in sample count as the disease severity ratio increased, suggesting that leaves with mild disease symptoms were more prevalent, whereas severely infected leaves were relatively fewer. Furthermore, distinct changes in the slope of the KDE curve were observed at disease severity ratios of 0.10, 0.35, and 0.55, signifying notable variations in the rate of decline. Consequently, these points were selected as classification thresholds for disease severity levels. Further observations revealed that leaves within the same disease severity category exhibited consistent morphological and color characteristics. Leaves with mild infections typically retained a higher degree of greenness and had fewer lesions, whereas those with severe infections often exhibited symptoms such as chlorosis and wilting. Moreover, the number of severely diseased leaves was relatively low, possibly due to natural shedding or the removal of heavily infected leaves during field management.

Based on these findings, a classification standard for strawberry leaf disease severity was established (see Table 4), aiming to provide a scientific basis for disease assessment and monitoring, thereby optimizing disease control strategies.

In this study, 485 valid datasets containing angular leaf spot disease were selected as the validation target. Initially, disease severity was manually pre-classified based on the observed symptoms. Subsequently, automatic classification was performed for each severity level using an OpenCV-based algorithm. The predicted disease severity was then validated against the manually labeled severity to compute the model’s classification accuracy. The study’s results are presented in Table 5, where “Correct Grading” denotes the number of images correctly classified, “Sample” represents the total number of images in each category, and “Accuracy” is defined as the ratio of the former to the latter.

## 3. Discussion

This study proposed a dual-phase classification approach, integrating YOLOv11-CARAFE-SE for leaf detection and segmentation with OpenCV-based threshold segmentation for disease spot identification, which achieved the automated severity assessment of strawberry angular leaf spot disease. This study addressed an issue that consists of three main parts: leaf segmentation, disease spot segmentation, and disease severity classification. The results showed that the YOLOv11-CARAFE-SE model achieved exceptional performance in accurately detecting and segmenting diseased strawberry leaves under field conditions, providing a robust foundation for the subsequent disease spot segmentation. The OpenCV-based threshold segmentation method successfully differentiated disease spots from healthy tissue on segmented leaves, enabling the establishment of four distinct severity levels based on the proportion of diseased area to total leaf area. Based on this dual-phase classification approach, an excellent result in disease severity classification was achieved, representing a significant advancement in automated plant disease assessment. Significant improvements were achieved in mitigating background interference and reducing the computational load; however, limitations persist in the disease spot segmentation process, primarily due to the influence of lighting conditions.

Specifically, under uniform illumination in diffuse lighting conditions, the HSV color space is generally considered more robust to changes in light intensity compared to RGB. However, it exhibits certain limitations under complex, non-uniform, and extreme lighting variations, such as backlighting and shadows. Although the vast majority of actual field photography occurs under uniform lighting, during the performance testing of our algorithm in this study, we considered some extreme cases. For instance, when shooting specifically under backlit conditions, green leaves often appear yellowish, resulting in a decrease in their H (hue) value, making them difficult to distinguish from areas with mild disease. This issue can be reasonably addressed by adjusting the shooting angle to avoid direct backlighting. When shooting under shadowed conditions, the H value remains relatively stable, while the S (saturation) and V (value) values decrease. Therefore, in our threshold segmentation algorithm, the threshold ranges for S and V were adjusted to be relatively wide, as long as it did not significantly compromise the segmentation performance. Nevertheless, these potential issues remind us that future research could incorporate algorithms such as adaptive thresholding and low-light image enhancement to better cope with extreme conditions.

At present, the research on strawberry diseases and insect pests is primarily focused on detecting various disease types, with relatively few studies addressing the assessment of disease severity [23]. For example, Nguyen et al. [24] proposed a strawberry leaf disease classification method based on multi-task U-Net for the detection of gray mold, powdery mildew, tip burn, and healthy leaves, where the model using a VGG16 backbone demonstrated the highest effectiveness, achieving a classification accuracy of 99.18%. Nguyen et al. [25] developed a model based on visual transformers that achieved the classification recognition of seven types of strawberry diseases through data augmentation and transfer learning techniques, reaching an accuracy of 92.7%. Karki et al. [26] studied the performance of different pre-trained models using transfer learning to identify various strawberry diseases in deep convolutional neural networks. The target diseases included angular leaf spot disease, anthracnose, gray mold, and powdery mildew on fruits and leaves. The results showed that ResNet-50 achieved the highest accuracy, reaching 94.4%. Kumar et al. [27] proposed a model that combined convolutional neural networks and support vector machines, featuring three convolutional layers, three max pooling layers, and a fully connected layer with ReLU for feature extraction. The CNN was used to identify discriminative features, which were then classified using an SVM classifier. The classification accuracy for strawberry leaf diseases reached 95%.

However, the existing disease identification technology has not yet solved the core demand in high-throughput phenotyping and precision breeding strategies for plant resistance research. By breeding different varieties of strawberries and studying the distribution of strawberry angular leaf spot disease, it is possible to screen strawberry varieties with disease-resistant traits. Therefore, the core task of this study is to identify the disease site and classify the degree of disease for strawberry angular leaf spot. Compared with other models, the accuracy of the proposed method is higher than most of the studies in Table 6. Part of the research on pest classification using deep learning and computer vision methods is shown in the table. In a recent study, Vats et al. [28] combined CNN’s analysis capabilities with federated learning for detecting different severity levels of tea plant diseases, achieving an accuracy rate of up to 97%. Liu et al. [29] utilized DeepLabV3+, PSPNet, and UNet to assess apple Alternaria leaf blotch severity across four levels (0: healthy, 1: mild, 2: moderate, 3: severe), achieving a 92.8% accuracy. Liu et al. [30] developed an application based on deep learning, where the underlying model used MobileNetV2-DeepLabV3 for leaf segmentation in the first stage and ResNet50-DeepLabV3 for lesion segmentation in the second stage. It achieved an average Intersection over Union (MIoU) of 98.65% for leaf segmentation and a 86.08% MIoU for lesion segmentation.

The results indicate that the developed algorithm shows considerable promise for accurately classifying the severity of strawberry angular leaf spots. Nevertheless, it is important to note that this study is preliminary; future research will therefore focus on developing algorithms that maintain their effectiveness even when applied to datasets more representative of diverse, real-world field conditions. For instance, firstly, our data collection, primarily focused on the early vegetative growth stage, was based on evidence suggesting this is when young, rapidly expanding leaves are most susceptible to angular leaf spot, making the data valuable for understanding early disease characteristics and for breeding programs. However, this targeted approach means we did not systematically evaluate the disease’s appearance across distinctly different vegetation stages, which can vary and impact the model’s robustness. Secondly, while our general daylight data acquisition proved effective, we did not specifically target potentially optimal but narrower observation windows, such as early morning with dew or after high humidity, which might enhance the visibility of certain bacterial symptoms. Thirdly, other vegetation conditions—like plant density, leaf wetness, or wind-induced movement—can significantly impact image quality and were not exhaustively controlled or analyzed in this initial phase. The subsequent phase of this study will address these challenges. Future work will focus on developing a portable device equipped with a built-in RGB camera that captures images in real time and transmits them wirelessly to a microcomputer. Following model processing, the device will provide real-time evaluations of disease severity. A further development is to integrate the device into a mobile robot deployed in orchards. By evaluating the disease severity of strawberry leaves, the robot can precisely control the pesticide dosage. The device should be lightweight and low-cost. Furthermore, a remote detection and consultation system is planned to broaden the application of deep learning-based image segmentation technology in agriculture, thereby providing essential support for promoting agricultural green development, protecting the environment, and ensuring food safety.

## 4. Materials and Methods

### 4.1. Datasets

The experimental dataset was constructed using two data sources. The primary source consisted of 227 field-collected strawberry leaf images captured by our research team. These images were acquired during daylight hours (typically between 9:00 a.m. and 4:00 p.m.) to ensure adequate natural illumination and to represent typical field observation conditions. The data collection for this initial study focused primarily on strawberry plants at the early vegetative growth stage, as this is often when angular leaf spot becomes more prevalent and is thus more valuable for guiding the breeding of disease-resistant varieties. To enhance the sample’s diversity and improve the model’s generalizability, we included 410 angular leaf spot disease images from a publicly available dataset provided by Afzaal et al. of the Artificial Intelligence Lab at the Department of Computer Science and Engineering, Chungbuk National University, South Korea [31]. This dataset contains agricultural field images captured under natural lighting conditions, ensuring a high reliability. A key advantage stems from its collection in real-world fields and greenhouses, which inherently introduces significant variability—including diverse backgrounds, complex field conditions, and varying illumination. The dataset was formatted in YOLO and annotated for strawberry leaf segmentation using AnyLabeling (V0.4.10) software, which supports assisted annotation. To facilitate model training, the labeled images were divided into training and validation sets in a 7:3 ratio. Furthermore, following a 2:1 ratio between the size of the pre-augmentation validation set and the test set, 79 diseased images and 13 normal images, totaling 92 images, were selected as the test set data. Additionally, various data augmentation techniques were applied to improve the YOLOv11 model’s generalizability, diversify the training data, and mitigate overfitting. These techniques included brightness adjustments, horizontal flipping, the addition of noise, and image translation. Finally, the experimental dataset comprised 3185 strawberry leaf images, including 2665 diseased images and 520 healthy images, as shown in Table 7.

### 4.2. Strawberry Leaf Segmentation Method Based on Improved YOLOv11

#### 4.2.1. YOLOv11

Given that the direct application of OpenCV-based image processing to raw field-collected images is prone to environmental variations such as illumination fluctuations, occlusions, and background noise [32,33], this study employed an improved YOLOv11 model to segment individual strawberry leaves. YOLO (You Only Look Once), introduced by Joseph Redmon and Ali Farhadi et al. in 2016 [34], is an object detection system that utilizes a single neural network. Building upon object detection, YOLOv5 [35] introduces a semantic segmentation functionality derived from target detection. Through iterative optimization, YOLOv11 achieves the following key innovations in both its model architecture and training strategies.

**Figure 6:**
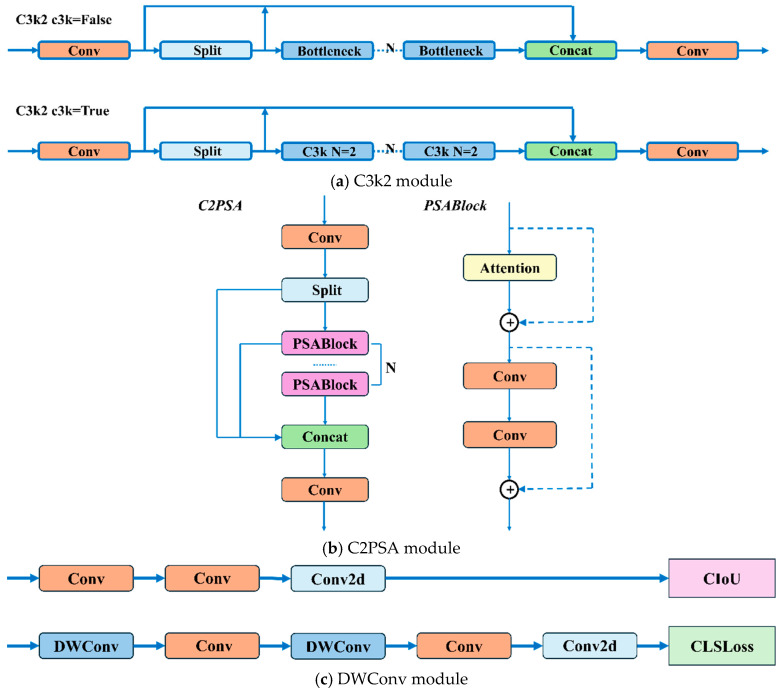
Three key innovations in YOLOv11 model architecture: C3k2 module (**a**), C2PSA module (**b**), and DWConv module (**c**).

(1) The C3k2 module is an enhanced design derived from the traditional C3 module. It provides enhanced feature extraction capabilities by integrating variable convolutional kernels and channel separation strategies. In the shallow layers of the network, when the c3k parameter is set to False, the C3k2 module becomes functionally equivalent to the standard C2f module. When the c3k parameter is set to True, the Bottleneck module is replaced with the C3 module, as illustrated in Figure 6a;
(2) The proposal of the C2PSA mechanism integrates a multi-head attention mechanism within the C2 framework. This mechanism is cascaded after the spatial pyramid fast pooling (SPPF) module, as illustrated in Figure 6b;
(3) The classification detection head within the original decoupled head has been enhanced by incorporating two depthwise separable convolutions (DWConvs), resulting in two DWConv layers in total. This modification significantly reduces both parameter count and computational complexity, as shown in Figure 6c;
(4) Significant modifications were made to the model’s depth and width parameters. Furthermore, YOLOv11 offers multiple variants with different scaling factors, allowing the flexibility to meet diverse requirements. In this experiment, the YOLOv11n model was chosen as the base model for further improvements due to its lower parameter count and faster inference speed, making it particularly well suited for deployment in embedded agricultural equipment scenarios. To facilitate the comparison of models, we incorporated YOLOv8 [36], YOLOv9 [37], and YOLOv10 [38].

#### 4.2.2. SE Attention

**Figure 7:**
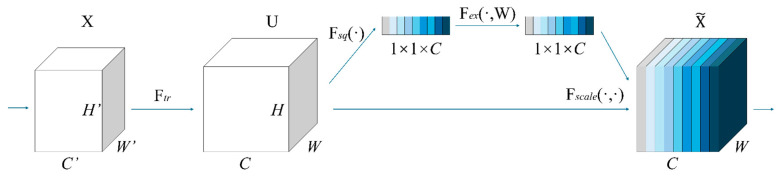
The specific structure of SE Attention.

In the detection and segmentation of strawberry diseased leaves, the complex background environment may cause substantial interference. Moreover, the lesions characteristic of strawberry angular leaf spot occupy only a small fraction of the image, thereby reducing the accuracy of disease detection and leaf segmentation. To address this challenge, incorporating attention mechanisms emerges as a promising approach to help the model isolate target regions containing critical information from a multitude of irrelevant background areas. In this study, the SE Attention mechanism [39] was employed. Additionally, to further assess the effectiveness of the SE attention mechanism, this study incorporated three distinct attention mechanisms—SE, CBAM [40], and Context Aggregation [41]. As illustrated in Figure 7, SE Attention is a typical channel attention mechanism. Let the input feature map be U ∈ R^(C × H × W). The improved attention generation process is as follows.

(1) Global statistics extraction: the channel description vector is obtained by applying mean pooling across spatial dimensions, as shown in Equation (1) [39]:
  (1) $$v_{C}=F_{sq}\left(u_{C}\right)=\frac{1}{H\times W}\sum_{i=1}^{H}\sum_{j=1}^{W}x_{C}\left(i,j\right)$$
  where $z_{C}$ represents the result of global average pooling for the C-th channel, H and W denote the height and width of the feature map, and $u_{C}\left(i,j\right)$ signifies the feature value at position $\left(i,j\right)$ in the C-th channel. The outcome is a vector $z=[z_{1},z_{2},\ldots ,z_{C}]$ of length C, which encapsulates the global information for each channel.
(2) Dynamic channel calibration: the gating mechanism is used to learn the nonlinear relationship between channels, as shown in Equation (2) [39]:
  (2) $$w=\sigma \left(W_{2}\cdot \delta \left(W_{1}\cdot v\right)\right)$$
  where $W_{1}\in R^{\frac{C}{r}\times C}$ is the weight matrix of dimension reduction, $W_{2}\in R^{C\times \frac{C}{r}}$ is the weight matrix of dimension increase, and the scaling factor r is the control parameter quantity.
(3) Feature recalibration: the learned channel weights are applied to the original feature map, as shown in Equation (3) [39]:
  (3) $${\tilde{x}}_{C}\left(i,j\right)=w_{C}\cdot x_{C}\left(i,j\right)$$
  where $s_{C}$ denotes the weight for the C-th channel, $X_{C}$ represents the C-th channel of the input feature map, and ${\tilde{X}}_{C}$ is the adjusted feature map. The output-weighted feature map is given by $\tilde{X}\in R^{C\times H\times W}$.

#### 4.2.3. CARAFE Module

CARAFE [42] is an upsampling method proposed to enhance feature maps in convolutional neural networks. Upsampling is commonly used to produce higher-resolution feature maps, which enables the network to capture more detailed information. In the context of the recognition of strawberry angular leaf spot disease in this study—where many lesions are small and challenging to detect—the improved upsampling method can effectively enhance the model’s performance. CARAFE leverages the underlying content information at each spatial location to predict reassembly kernels and subsequently reassembles the features within a predefined local neighborhood. By incorporating content-aware information, CARAFE deploys adaptive and optimized reassembly kernels at various spatial locations, thereby outperforming mainstream upsampling operators. The DySample upsampling method is a technique that leverages a dynamic sampling strategy to enhance the detailed representation of low-resolution feature maps. In the experiments, DySample was introduced for comparative analysis [43].

**Figure 8:**
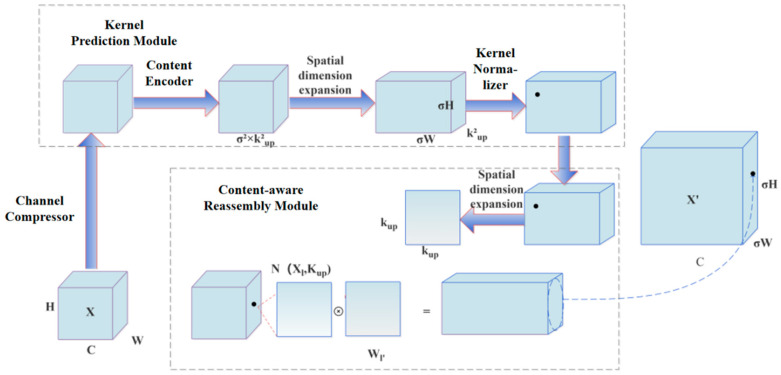
The specific structure of the CARAFE Module.

CARAFE consists of two main components—the kernel prediction module and the content-aware reassembly module—which operate in two sequential steps. It proceeds in two sequential steps. In the first step, a reassembly kernel is predicted for each target location. Subsequently, the predicted kernel is employed to reassemble the features. Given an input feature map of dimensions H × W × C and an upsampling factor σ, a new feature map of dimensions σH × σW × C is produced. Specifically, the kernel prediction module generates location-specific kernels based on the input feature content, which are subsequently applied by the content-aware reassembly module to reassemble the feature map. Figure 8 illustrates the fundamental framework of CARAFE.

#### 4.2.4. Proposed Model

**Figure 9:**
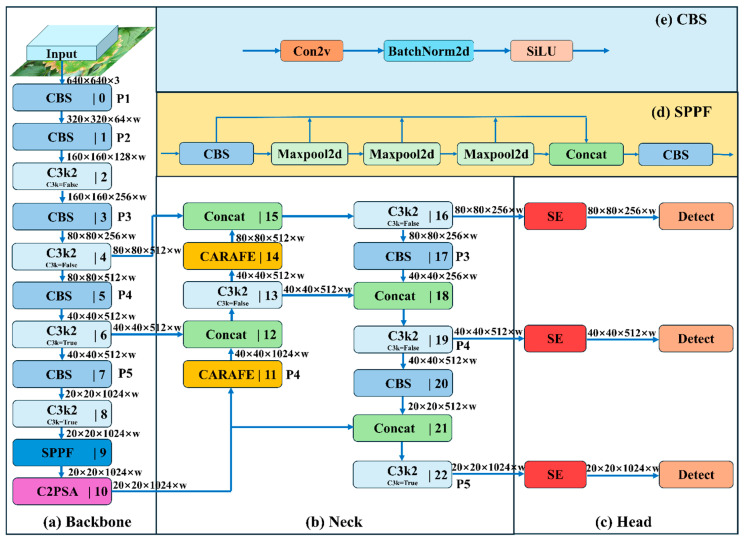
The proposed YOLOv11-CARAFE-SE network structure diagram: Backbone (**a**), Neck (**b**), Head (**c**), SPPF (**d**), and CBS (**e**).

The greenhouse cultivation environment is highly complex. Common challenges in leaf segmentation include similar textures between the target and background, target occlusion, and similarities among different target types. To enhance the model’s accuracy, this study optimized the YOLOv11 framework by integrating the SE and CARAFE modules, thereby improving the overall performance. Through extensive experimentation, the improved YOLOv11-CARAFE-SE model was developed by inserting an SE module between the Neck and Head and replacing the conventional upsampling module with the CARAFE module. Due to the fundamental similarities in strawberry leaf and angular leaf spot disease morphology across varieties, coupled with the enhanced feature learning capabilities imparted by these modifications, the improved YOLOv11-CARAFE-SE model is designed for robust and broad applicability in detecting angular leaf spot across different strawberry cultivars. The overall architecture of the improved YOLOv11-CARAFE-SE model is illustrated in Figure 9.

### 4.3. OpenCV-Based Lesion Segmentation Method and Disease Severity Grading

**Figure 10:**
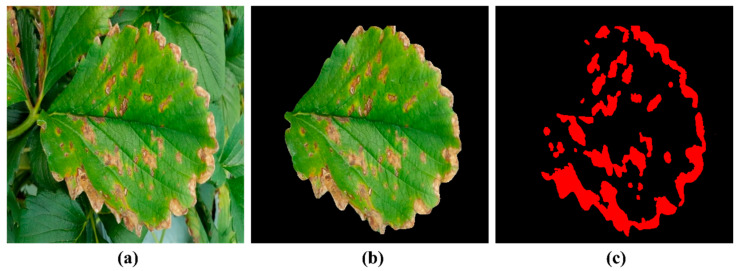
Difficult example of strawberry angular leaf spot labeling: (**a**) is the original image, (**b**) is the segmented leaf, and (**c**) is the result of labeling the ideal spot area, highlighting the difficulty of manual annotation.

Accurate disease grading in complex field environments presents significant challenges due to background interference and variations in leaves’ appearance (Figure 10a). To address this, this research first employs YOLO to extract individual leaf regions, effectively isolating them from the background (Figure 10b). However, precise lesion segmentation remains difficult due to the small, irregular, and numerous nature of the disease spots, which deep learning networks struggle to detect accurately [44]. Additionally, manual annotation for training such models is labor-intensive and costly (Figure 10c). Given the distinct color contrast between lesions and healthy leaf tissue, we leverage an OpenCV-based threshold segmentation method to efficiently extract lesion areas without the need for extensive training data, ensuring both accuracy and computational efficiency.

Diseased leaf images were selected from the dataset, and an enhanced YOLOv11 segmentation model was employed to generate high-quality binarized mask images. In the resulting masks, white pixels accurately delineated the leaf regions, whereas black pixels indicated the background. By applying a bitwise AND operation to the mask and the original image, a segmented color image of the diseased leaf was obtained. By leveraging the model’s robustness in complex scenarios—including multi-scale feature fusion and adaptive noise suppression—the segmentation boundaries remained sharp and accurate even under challenging conditions, such as uneven lighting or sensor noise, thereby eliminating the need for additional post-processing steps.

The processed leaf images were converted from the RGB color space to the HSV color space. This transformation facilitated threshold segmentation in the subsequent steps. The HSV color space consists of three components: hue (H), saturation (S), and value (V). The HSV color space, especially its H component, is characterized by its relative robustness to changes in light intensity under diffuse lighting conditions with uniform illumination, a property not as strongly observed in the RGB color space. Thus, threshold setting primarily focused on adjusting the H component. Extensive experiments, guided by empirical testing and the research objectives, were conducted to determine an optimal threshold value, as summarized in Table 8:

The proportion of the diseased area relative to the total leaf area was employed. Accordingly, strawberry angular leaf spot was classified into distinct severity levels. After validating the effectiveness of the lesion segmentation algorithm, the entire dataset was analyzed, and classification thresholds were established based on the distribution characteristics of this ratio. The calculation is given by Equation (4) [45]:

(4) $$P=\frac{S_{D}}{S_{L}}$$

where $S_{L}$ denotes the total area of the strawberry leaf, $S_{D}$ denotes the segmented diseased area, and P is the proportion of the diseased area to the total leaf area.

**Figure 11:**
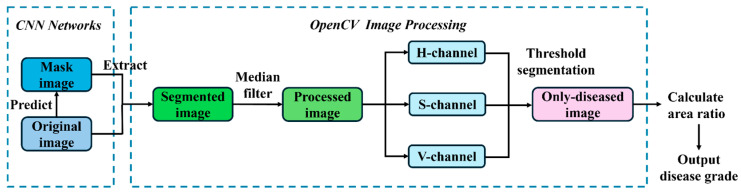
The research flowchart of this study includes CNN networks to segment individual strawberry leaves, OpenCV image processing to segment the diseased part of the spot, and the output of disease grades.

The severity classification of strawberry angular leaf spot was conducted in dual phases. In the first phase, each input image was processed by a pre-trained YOLO model for binary classification. If disease-free leaves were detected, they were directly classified as healthy samples; conversely, an infected leaf image was further processed by the trained segmentation model to generate a prediction mask, which was subsequently fused with the original image and preprocessed. In the second phase, for the positive samples identified by the YOLO model, an OpenCV-based threshold segmentation algorithm was applied to compute the ratio of the diseased area to the total leaf area, which was then used to determine the severity level. The research flowchart is illustrated in Figure 11. The pre-screening function of the YOLO model effectively reduced the computational burden.

### 4.4. A Detection Platform for Strawberry Angular Leaf Spot Severity Based on PyQt5

**Figure 12:**
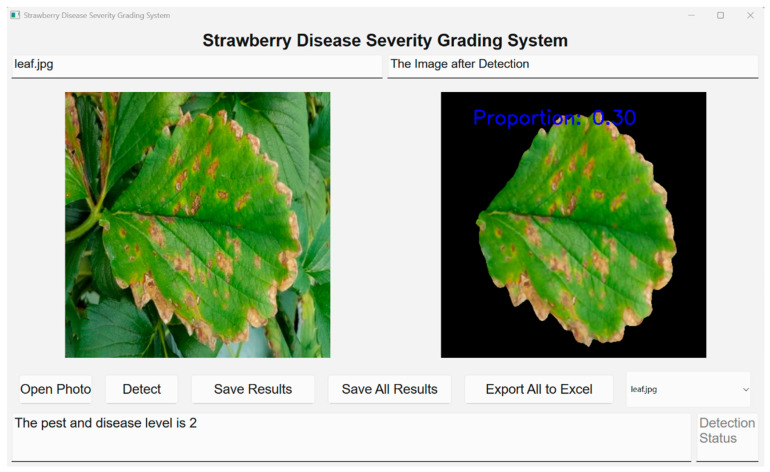
A PyQt5-based software application designed for strawberry angular leaf spot disease classification.

To implement the improved YOLOv11-CARAFE-SE model in practical applications, this study developed an efficient and user-friendly software application based on PyQt5 for assessing the severity of strawberry angular leaf spot, as depicted in Figure 12. The software’s user interface was designed using PyQt5 to create an intuitive graphical user interface (GUI), and the developed model, along with its runtime environment, was packaged using the PyInstaller tool. Upon launching the software, users could select images from a designated folder for analysis. By clicking the “Start Detection” button, the software initiated either single-image or batch detection. The detection results are displayed on the interface, with the corresponding disease severity level automatically generated. Furthermore, users have the option to review the detection results for each image individually. Finally, by clicking the “Export to Excel” button, the results could be exported in Excel format.

### 4.5. Equipment

The entire process of model training and validation was conducted on a personal computer (CPU: Intel^®^ Core™ i9 14900K @6.00 GHz; GPU: NVIDIA GeForce RTX 4080 16G). The environment was configured as shown in Table 9. The training environment was built using PyTorch (V2.5.0), with the GPU parameters shown in Table 10, including an input image size of 640 × 640 pixels, a batch size of 16, and a training duration of 200 epochs. The maximum learning rate was set to 0.001, and the optimizer used was Adaptive Moment Estimation (Adam) [46]. A weight decay of 0.0005 was applied to mitigate overfitting. The training process was executed with 32 threads to enhance computational efficiency. The final set of hyperparameters, which demonstrably yielded the best performance in this study, was established through a meticulous and iterative process of adjustments. Each selection was systematically evaluated and validated against performance on a dedicated validation set, thereby confirming this configuration as the optimal one achieved.

### 4.6. Model Evaluation

To comprehensively evaluate the performance of the model, a set of evaluation metrics—precision (P), recall (R), mean average precision (mAP), and inference time—was selected. Among them, P measures the proportion of samples predicted as positive that are indeed true positives, as defined in Equation (5) [47]. R represents the proportion of actual positive samples that are correctly identified by the model, as shown in Equation (6) [47]. Average precision (AP) provides a comprehensive assessment based on both precision and recall by calculating the area under the precision–recall (P-R) curve. mAP is obtained by averaging the AP values across all classes, as expressed in Equation (7) [47]. In particular, mAP@0.5 represents the average precision at an IoU threshold of 0.5, while mAP@0.5:0.95 is calculated by averaging the AP over multiple IoU thresholds, ranging from 0.5 to 0.95, thereby providing a more comprehensive evaluation of the model’s performance. A higher mAP indicates superior performance. Furthermore, in the context of image segmentation, the four metrics, P, R, mAP@0.5, and mAP@0.5:0.95, are computed separately at both the box level, which denotes the approximate location and category of the object, and the mask level, which delineates the pixel-level boundary of the target. Inference time is defined as the duration required for the model to perform inference on a single image; a lower inference time indicates a faster processing speed [48].

(5) $$Precision=\frac{TP}{TP+FP}$$

(6) $$Recall=\frac{TP}{TP+FN}$$

(7) $$mAP=\frac{\sum_{i=1}^{C}AP_{i}}{C}$$

where *TP*, *FP*, *FN*, and *TN* stand for true positive, false positive, false negative, and true negative, respectively. The ${AP}_{i}$ is the average precision value at the i-th species. C is the total number of species.

## 5. Conclusions

This research addressed the challenge of accurately evaluating strawberry angular leaf spot disease severity in complex environments by developing a dual-phase classification method integrating YOLOv11 with OpenCV-based algorithms. The improved YOLOv11-CARAFE-SE model enhanced the detection performance (box mAP@0.5 increased from 91.8% to 93.2%, mask mAP@0.5 from 92.1% to 93.0%) while significantly reducing the inference time by 30.8% (from 1.3 ms to 0.9 ms) and computational requirements from 10.3 to 10.1 GFLOPS. The second phase, introducing an OpenCV-based threshold segmentation algorithm with a disease severity classification standard, bridges the gap between detection and practical disease assessment. Furthermore, this dual-phase grading framework could achieve an average accuracy of 94.2% in detecting selected strawberry angular leaf spot samples. These technical advances enable broader deployment on accessible hardware in field conditions. Beyond its technical contributions, this methodology advances sustainable agriculture by facilitating the breeding of disease-resistant varieties, ultimately contributing to reduced economic losses and improved food security, while demonstrating how deep learning and computer vision techniques can be effectively integrated for agricultural applications.

## Figures and Tables

**Table 1:** Comparative results of ablation experiments on the test set.

Methods	Box			Mask			Inference Time/ms	GFLOPS
	Precision /%	Recall /%	mAP@0.5 /%	Precision /%	Recall /%	mAP@0.5 /%		
YOLOv8	86.1	84.6	91.1	86.2	84.7	91.0	1.1	12.0
YOLOv9	88.1	81.2	91.0	88.5	80.5	90.3	1.9	53.2
YOLOv10	92.5	82.2	91.7	91.5	82.9	91.9	0.9	10.6
YOLOv11	88.1	86.0	91.8	88.4	86.3	92.1	1.3	10.3
YOLOv11-SE	86.6	87.6	92.3	86.6	87.6	92.6	1.2	10.3
YOLOv11-CARAFE	89.2	86.0	93.0	89.2	86.0	93.0	0.9	10.1
YOLOv11-CARAFE-SE	88.3	87.2	93.2	88.2	87.3	93.0	0.9	10.1

**Table 2:** Comparative experiment for different attention mechanisms.

Methods	Box			Mask			GFLOPS
	Precision/%	Recall/%	mAP@0.5/%	Precision/%	Recall/%	mAP@0.5/%	
YOLOv11 (No Attention)	88.4	86.0	91.9	88.7	86.2	92.0	10.3
SE	86.7	87.6	92.5	86.7	87.6	92.6	10.4
CBAM	91.2	82.0	92.3	91.1	81.9	91.8	10.4
Context Aggregation	90.1	84.0	92.0	91.4	83.8	91.8	10.4

**Table 3:** Comparative experiment for different upsampling methods.

Methods	Box			Mask			GFLOPS
	Precision/%	Recall/%	mAP@0.5/%	Precision/%	Recall/%	mAP@0.5/%	
YOLOv11 (nearest)	88.3	85.8	91.8	88.7	86.0	92.0	10.3
YOLOv11 (bilinear)	90.9	80.5	91.6	90.8	80.5	91.7	10.3
CARAFE	89.3	86.1	93.1	89.3	86.1	93.1	10.1
DySample	89.1	83.9	91.8	89.4	84.1	92.3	10.4

**Table 4:** Strawberry angular leaf spot disease severity classification standard proposed in this study, defining four disease levels based on thresholds of 0.1, 0.35, and 0.55.

Severity Level	Symptoms	Disease Area Ratio	Data Quantity
Level 1	Small water-soaked spots visible on the back of the leaf	(0, 0.10]	138
Level 2	Spot area expands, leaf edges appear dried and dehydrated	(0.10, 0.35]	217
Level 3	Large disease spots appear but do not completely merge to cover the entire leaf	(0.35, 0.55]	69
Level 4	Most of the leaf area is covered with red-brown lesions, which merge into a large patch	(0.55, 1]	61

**Table 5:** Performance of overall verification, showing achieved accuracy.

Severity Level	Correct Grading	Sample	Accuracy (%)
Level 1	134	139	96.4
Level 2	192	208	92.3
Level 3	70	75	93.3
Level 4	61	63	96.8
Total	457	485	94.2

**Table 6:** The comparison results of different models proposed by various teams for plant disease severity grading research.

References	Plants	Model	Disease Types/Levels	Accuracy
Nguyen et al. [24]	Strawberry	MT-UNet (VGG16 backbone)	Gray Mold, Powdery Mildew, Tip Burn, Healthy	98.9%
Nguyen et al. [25]	Strawberry	Vision Transformer	Anthracnose Fruit Rot, Flower Blight, Gray Mold, Leaf Spot Disease, Powdery Mildew On Leaves, Powdery Mildew On Fruits	92.7%
Karki et al. [26]	Strawberry	Resnet-50	Angular Leaf Spot, Anthracnose, Gray Mold, and Powdery Mildew on Both Fruit and Leaves	94.4%
Kumar et al. [27]	Strawberry	CNN-SVM	Powdery Mildew, Leaf Scorch, Leaf Blight	95.0%
Vats et al. [28]	Tea	CNN	(1_V Low) 1–20%, (2_Low) 21–40%, (3_Med) 41–60%, (4 High) 61–80%, (5_V High) 81–100%	97.0%
Liu et al. [29]	Apple	DeepLabV3+, PSPNet, UNet	0 (Healthy), 1 (Mild), 2 (Moderate), 3 (Severe)	92.8%
Liu et al. [30]	Wheat	MobileNetV2-DeepLabV3+ + ResNet50-DeepLabV3+	IoU score based on health category (IoU-H)	86.08%
Proposed method	Strawberry	YOLOv11-based	0 (Healthy), 1 (0, 10%], 2 (10%, 35%], 3 (35%, 55%], 4 (55%, 100%]	94.2%

**Table 7:** Strawberry angular leaf spot disease dataset.

Angular Leafspot	Training Images	Val Images	Test Images	Total Images
Diseased	1865 (×5)	800 (×5)	79	2744
Healthy	360 (×5)	160 (×5)	13	533

**Table 8:** The optimal threshold values for segmentation in the HSV color space in this segmentation task.

	H_max	H_min	S_max	S_min	V_max	V_min
Diseased	37	1	210	30	244	100
Healthy	64	38	255	100	200	57

**Table 9:** Configuration of the training environment.

Name	Information
CPU	Intel^®^ Core™ i9 14900K @6.00 GHz
GPU	NVIDIA GeForce RTX 4080 16G
Operating System	Windows 11
Deep Learning Framework	Pytorch 2.5.0
Programming Language	Python 3.12.7
Integrated Development Environment	VScode 1.92
Package Management Tools	Anaconda 2.5.2

**Table 10:** Experimentally determined optimal training parameter settings for YOLOv11-based deep learning models.

Hyperparameter	Value
Input image size	640 × 640
Batch size	16
Epoch	200
Maximum learning rate	0.001
Optimizer	AdamW
Weight decay	0.0005
Thread count	32

## Data Availability

Data are available on request due to privacy. The YAML configuration file for the proposed YOLOv11-CARAFE-SE model, used for training and evaluation, is publicly available in the Mendeley Data repository at https://doi.org/10.17632/R8K5ZZTJCM.1.
